# Cognitive behavioral treatment for irritable bowel syndrome: a recent literature review

**DOI:** 10.1186/s13030-021-00226-x

**Published:** 2021-11-27

**Authors:** Nagisa Sugaya, Kentaro Shirotsuki, Mutsuhiro Nakao

**Affiliations:** 1 Unit of Public Health and Preventive Medicine, School of Medicine, Yokohama City University, 3-9 Fukuura, Kanazawa-ku, Yokohama City, 236-0004 Japan; 2 Graduate School of Human and Social Sciences, Musashino University, Tokyo, Japan; 3 Department of Psychosomatic Medicine, School of Medicine, International University of Health and Welfare, Chiba, Japan

**Keywords:** Irritable bowel syndrome, Cognitive behavior therapy, Psychological assessment, Brain-gut axis

## Abstract

Irritable bowel syndrome (IBS) is a gastrointestinal psychosomatic disorder that often develops and worsens with stress. Hence, it is important to treat it from both, its physical and mental aspects. We reviewed recent research on cognitive-behavioral therapy (CBT)—one of the most widely studied psychological treatments for IBS—since it focuses on addressing the cognitions and behaviors associated with IBS symptoms, and combines diverse content, such as cognitive techniques, exposure, stress management, and mindfulness, whose effects have been widely studied. Research on CBT for IBS varies not only in terms of content of the interventions, but also in terms of implementation (individual or group, face-to-face or online). Internet-delivered CBT has recently shown the possibility of providing more accessible and cost-effective psychological intervention to IBS patients in formats, other than face-to-face. In recent years, many standardized scales that allow for IBS-specific psychological assessments have been used in clinical studies of CBT for IBS. Tools that competently deliver effective interventions and properly measure their effectiveness are expected to spread to many people suffering from IBS.

## Introduction

Irritable bowel syndrome (IBS) is one of the most common functional gastrointestinal disorders. Since its prevalence in the general population is as high as 5–10% [1], its associated medical and economic problems have also been pointed out [2]. The Rome IV criteria [3], the diagnostic criteria currently used internationally, defines IBS as recurrent abdominal pain occurring on an average, at least 1 day per week in the last 3 months, and associated with at least two of the following three abnormalities: (1) defecation-related, (2) change in defecation frequency, or (3) change in stool characteristics (appearance). The pathogenesis of IBS includes: (1) abnormalities in gastrointestinal motility, (2) lowered gastrointestinal sensory threshold, and (3) psychological abnormalities (anxiety, depression, etc.). The pathophysiology of IBS includes: (1) abnormalities in gastrointestinal motility, (2) decreased gastrointestinal sensory threshold, and (3) psychological abnormalities (anxiety, depression, etc.), all of which, are related to abnormalities in the functional relationship between the brain and intestine (brain-gut connection). Since IBS is a gastrointestinal psychosomatic disorder that often develops and worsens with stress, it is important to treat it from both the physical and mental aspects [4]. We shall review recent research on cognitive-behavioral therapy—one of the most widely studied psychological treatments for IBS.

### Application of cognitive behavioral therapy for IBS

Guidelines for the treatment of IBS [4] recommend psychological interventions for patients, who do not respond to standard pharmacotherapy. Psychological interventions, such as cognitive behavioral therapy (CBT), relaxation, hypnotherapy, and dynamic psychotherapy have been reported to be effective for treating IBS [5]. Since it has been reported that cognitions and behaviors associated with IBS symptoms are involved in unpleasant emotions, such as anxiety and abdominal symptoms [6, 7], CBT targeting cognitions and behaviors associated with such symptoms have been widely tested for efficacy. Toner et al. [8] stated that the purpose of CBT in IBS is to reconfigure how IBS is viewed, given that, its purpose is to help patients to: (1) reframe their view of IBS from helplessness and hopelessness to resourcefulness and hopefulness, (2) identify the relationship between their thoughts, feelings, and behaviors with the environment and IBS symptoms, and (3) identify and implement more effective coping strategies to improve the quality of life (QOL). A recent meta-analysis reported that IBS patients’ pretreatment cognitive-emotional characteristics (comorbidity of mood or anxiety disorder, symptom catastrophizing and worries, tendencies of somatosensory amplification, low symptom acceptance, or self-efficacy) predict bad patient outcomes in CBT [9].

### Various forms of CBT for IBS

CBT for IBS focuses on addressing the cognitions and behaviors associated with IBS symptoms and combines diverse content, such as cognitive techniques, exposure, stress management, and mindfulness, the effects of which, have been widely studied. For example, CBT for IBS conducted in a previous study by Lackner et al. [10] consisted of the provision of information regarding brain-gut interactions; self-monitoring of gastrointestinal symptoms, their antecedents (i.e., triggers) and consequences; muscle relaxation to dampen physiological arousal and increase control over gastrointestinal symptoms; worry control to challenge and dispute negatively skewed thinking patterns; flexible problem solving to aid in the deployment of more effective ways of managing realistic stressors; and relapse prevention training to maintain treatment gains. In addition, CBT combined with exposure has been reported to be effective, especially in patients with significant avoidance behaviors related to IBS symptoms [11]. Several studies have also demonstrated the effectiveness of CBT combined with mindfulness [12], and the addition of exposure has been reported to increase IBS symptoms and related anxiety as well as QOL improvements [13]. Recently, several studies have demonstrated the efficacy of CBT with interoceptive exposure in IBS patients [14, 15]. Interoceptive exposure involves exposing one’s self to self-induced abdominal sensations (e.g., tightening abdominal muscles, consuming foods that are to be avoided, etc.) to reduce anxiety in response to abdominal disturbance common in IBS [15].

Research on CBT for IBS varies not only in terms of the interventions’ contents, but also in terms of its implementation (individual or group, face-to-face or online). Research on internet-delivered CBT has been increasing in recent years (Table 1), and its effects on IBS and functional abdominal pain have been seen not only in adults, but also in children [16–19]. CBT delivered through the telephone and internet has been shown to be effective, suggesting the usefulness of conducting it remotely [20]. Furthermore, internet-delivered CBT has been reported to be effective not only in improving abdominal pain, QOL, and psychological symptoms, but also in reducing health care costs [18], and is expected to become more widespread in the future. Home and clinic-based CBT have been reported to result in substantial and enduring relief of multiple IBS symptoms for patients with treatment-refractory IBS, and its effects generally extended post treatment, for a 12-month period [10]. CBT with minimal medical visits has also been shown to be cost-effective [21]. Therefore, if CBT—conducted at home via the internet or other means, as well at medical institutions—becomes widespread, it may make it easier for IBS patients, who have difficulty visiting medical institutions frequently to access appropriate and cost-effective treatments. With the use of internet or telephone-delivered CBT, however, we should be prepared with countermeasures if symptoms worsen. For example, for a child patient, a guardian should accompany and then monitor the child closely. A hotline should be set up in case of emergency.

**Table 1:** Recent research on the effect of internet-delivered CBT for IBS patients

Author	Year	Participants	Interventions	Results
Bonnert et al	2017	Internet-CBT group: *N* = 47 Waitlist control group: *N* = 54 Age range: 13–17 years.	The internet-CBT spanned over 10 weeks and included 10 weekly modules directed at adolescents, and five modules directed at parents. The main principle of the treatment was to use exposure exercises to reduce symptom-fear and avoidance. The modules consisted of short texts, examples, audio-files, and videos. Clinical psychologists with CBT-training provided online support to adolescents and parents.	1) Pretreatment to post-treatment changes on gastrointestinal symptoms as the primary outcome and on almost all secondary outcomes was significantly larger for the internet-CBT group as compared with the control group. 2) After 6 months, the results were stable or significantly improved.
Lalouni et al	2019	Internet-CBT group: *N* = 46 Treatment as usual group: *N* = 44 Age range: 8–12 years.	The children’s exercises included exposure to symptom-provoking stimuli (e.g., eating certain foods) and situations in which they feared having symptoms (e.g., physical exercise), and those that decreased their symptom-controlling strategies (e.g., precautionary toilet visits). A short mindfulness exercise was taught as a means to increase awareness of the abdominal symptoms. The parents encouraged their children to work on exposure exercises that decreased their attention from symptom complaints, and increased their attention toward more adaptive behaviors. Licensed psychologists who specialized in CBT provided online support to children and their parents.	1) Gastrointestinal symptom severity, quality of life, gastrointestinal-specific anxiety, avoidance behaviors, and parental responses to children’s symptoms improved significantly in the internet-CBT group, as compared with the group that received treatment as usual . 2) The effects of internet-CBT persisted through 36 weeks of follow-up evaluation. 3) The internet-CBT group had significantly less health care usage than group that received treatment as usual, with an average cost difference of US $137.
Hughes et al	2020	Internet-CBT group: *N* = 17 Telephone-CBT group: *N* = 17 Mean age (SD): 39.94 (11.71) years.	Internet-CBT program consisted of eight web-based sessions to be completed on a weekly basis, three 30-min telephone support sessions over 9 weeks. The telephone-CBT group received six one-hour telephone-based CBT sessions over 9 weeks. The same CBT content was delivered via telephone and website, with only the mode of delivery being different. The CBT content was based on an empirical cognitive behavioral model of IBS, and comprised educational, behavioral, and cognitive techniques aimed at improving bowel habits, developing stable healthy eating patterns, addressing unhelpful thoughts, managing stress, reducing symptom that focused on preventing relapse. Trained CBT therapists provided telephone support.	(Inductive thematic analysis was used to analyze the data). 1) Participants liked the flexibility of CBT via internet and telephone-, and this facilitated engagement. 2) Potential barriers to engagement in both the groups included: initial skepticism and concerns about the biopsychosocial nature of CBT; initial concerns about telephone-delivered talking therapy; challenges of maintaining motivation and self-discipline given their already busy lives, and finding nothing new in the internet-CBT. 3) Participants described helpful changes in their understanding of IBS, attitudes toward IBS, ability to recognize IBS patterns, and IBS-related behaviors. 4) Consistent with the trial results, participants described lasting positive effects on their symptoms, work, and social lives.
Hunt et al	2021	Internet-CBT group: *N* = 62 Waitlist control group: *N* = 59 Age range: 18–63 years.	The application program of internet-CBT consists of eight weekly modules focusing on psychoeducation, relaxation training, exercise, the cognitive model of stress management, applying CBT to IBS symptoms, reducing avoidance through exposure therapy, behavioral experiments, and information about diet.	Gastrointestinal symptoms, IBS-related quality of life and cognition, visceral sensitivity, and fear of foods improved significantly in the internet-CBT group.
Owusu et al	2021	Internet-CBT group: *N* = 25 Control group: *N* = 11 Age range: 19–61 years.	The internet-CBT program is self-paced. It consists of eight sessions, designed to be completed within 12 weeks. Therapeutic targets include: knowledge of the biopsychosocial model of IBS and the brain-gut connection; improving bowel habits; developing a stable eating routine; reducing hypervigilance to IBS symptoms; identifying unhelpful gastrointestinal-related thoughts; recognizing how these automatic thoughts affect behaviors, emotions, and gastrointestinal symptoms; and accepting and processing emotions. The internet-CBT program delivers cognitive and behavioral skills and techniques in an interactive manner through repetition of brief learnings and exercises.	1) IBS symptom severity significantly improved at 2-month and 3-month follow-up. The within-group effect size between baseline and 3-month follow-up gastrointestinal symptom scores was large and 63.6% experienced a clinically meaningful improvement. 2) Gastrointestinal-specific anxiety symptoms and cognitions significantly improved at 2-month follow-up, as did unhelpful IBS safety behaviors. 3) Clinically meaningful improvement was observed in depressive and anxiety symptoms at 3-month follow-up among participants with symptoms above the clinical threshold at baseline.

*CBT* cognitive behavior therapy, *IBS* irritable bowel syndrome

### Psychological assessment tools of CBT for IBS

Psychological interventions to alter cognition and behaviors specific to IBS, require appropriate psychological assessment. Some of the scales commonly used in recent research on CBT for IBS are presented below.

#### Visceral sensitivity index

The Visceral Sensitivity Index is a unidimensional 15-item scale that measures gastrointestinal symptom–specific anxiety (e.g., “I often fear that I will not be able to have normal bowel movements”) [22]. Items are rated on a Likert scale ranging from 0 (strongly disagree) to 5 (strongly agree). It has a high internal consistency (Cronbach’s α = 0.93) [23]. Its qualitative score was classified as: 0–10 (minimal or mild), 11–30 (moderate), and 31–75 (severe).

#### Cognitive scale for functional bowel disorder

The Cognitive Scale for Functional Bowel Disorder [8] includes 25 items that measure maladaptive cognition related to abdominal symptoms (e.g., “I am always sick with bowel problems”). The items are rated from 1 (strongly disagree) to 7 (strongly agree). Its total score ranges from 25 to 175, and it has a high internal consistency (Cronbach’s α = 0.93).

#### Gastrointestinal cognitions questionnaire

The Gastrointestinal Cognitions Questionnaire consists of 16 self-report items rated on a 5-point Likert scale, ranging from 0 (hardly) to 4 (very much). Individual items are summed, and the total scores range from 0 to 64. The questionnaire consists of three subscales: the pain/life interference subscale (e.g., “When I feel my GI symptoms acting up, I am afraid the pain will be excruciating and intolerable”), the social anxiety subscale (e.g., “If I have to get up and leave an event, meeting, or social gathering, to go to the bathroom, people will think there is something wrong with me”), and the disgust sensitivity subscale (e.g., “The thought of fecal incontinence is terrifying. If it happened, it would be awful”). The scale has been shown to have good internal consistency (Cronbach’s α = 0.92) [24]. Its qualitative score was classified as: 0–19 (minimal or mild), 20–39 (moderate), and 40–64 (severe).

#### Irritable bowel syndrome-behavioral responses questionnaire

The Irritable Bowel Syndrome-Behavioral Responses Questionnaire [25] includes 26 items with responses ranging from 1 (never) to 7 (always), and its total score ranges from 26 to 182. It consists of two factors: avoidance behavior scale (e.g., “I avoid going out in case I have problems with my IBS”) and control behavior scale (e.g., “After opening my bowels, I check my stool for abnormalities”), and has sufficient internal consistency (Cronbach’s α = 0.86).

#### The irritable bowel syndrome quality of life questionnaire

The Irritable Bowel Syndrome Quality of Life questionnaire is a 34-item self-report measure, specifically designed to assess the impact of IBS on QOL (e.g., “I am bothered by how much time I spend on the toilet”) [26, 27]. It has a high internal consistency (Cronbach’s α = 0.95) [27]. Its qualitative score was classified as: 0–31 (minimal or mild), 32–66 (moderate), and 67–100 (severe impairment).

### The effect of CBT for IBS and its psychobiological mechanism

A number of previous studies have demonstrated that CBT improves anxiety, depression, QOL, and abdominal symptoms in patients with IBS [28–30]. It has also been reported that CBT reduces neural activity in the parahippocampal gyrus and lower right cingulate cortex, and that changes in limbic activity are associated with improvements in abdominal symptoms and anxiety [31]. These brain regions are associated with excessive vigilance and emotional memory [32]. Some previous studies reported that CBT has a direct effect on IBS symptoms, that were independent of its effect on psychological distress, suggesting that the reduction of IBS symptoms improve psychological distress, rather than vice versa [33]. Furthermore, a meta-analysis of CBT for IBS reported more efficacy for abdominal symptoms rather than psychological symptoms [30]. In other words, it is interesting to note, that cognitive and behavioral transformation in IBS may contribute to the improvement of abdominal symptoms through direct involvement in the brain-gut correlation process of IBS, and thus alleviate psychological distress, such as anxiety and depression.

## Conclusion

We reviewed recent research on the characteristics and effects of CBT for IBS, its mechanism, and tools for measuring its effectiveness. The biopsychological mechanisms underlying the effects of CBT have been gradually elucidated in parallel with the elucidation of the biological characteristics of IBS. In addition, internet-delivered CBT has recently shown the possibility of providing more accessible and cost-effective psychological interventions to IBS patients, in formats other than face-to-face. These tools are expected to spread to many IBS patients, including those, suffering from IBS, but who do not visit medical institutions.

## Data Availability

Not applicable.

## Abbreviations

IBS: Irritable bowel syndrome
CBT: Cognitive behavior therapy
QOL: Quality of life

## References

[1] Palsson OS, Whitehead WE, Van Tilburg MAL, Chang L, Chey W, Crowell MD (2016). Development and validation of the Rome IV diagnostic questionnaire for adults. Gastroenterology..

[2] Black CJ, Ford AC (2020). Global burden of irritable bowel syndrome: trends, predictions and risk factors. Nat Rev Gastroenterol Hepatol.

[3] Drossman DA (2016). Functional gastrointestinal disorders: history, pathophysiology, clinical features, and Rome IV. Gastroenterology..

[4] Fukudo S, Okumura T, Inamori M, Okuyama Y, Kanazawa M, Kamiya T (2021). Evidence-based clinical practice guidelines for irritable bowel syndrome 2020. J Gastroenterol.

[5] Ford AC, Lacy BE, Harris LA, Quigley EMM, Moayyedi P (2019). Effect of antidepressants and psychological therapies in irritable bowel syndrome: an updated systematic review and meta-analysis. Am J Gastroenterol.

[6] Sugaya N, Nomura S, Shimada H (2012). Relationship between cognitive factors and anxiety in individuals with irritable bowel syndrome. Int J Behav Med.

[7] Windgassen S, Moss-Morris R, Goldsmith K, Chalder T (2019). Key mechanisms of cognitive behavioural therapy in irritable bowel syndrome: the importance of gastrointestinal related cognitions, behaviours and general anxiety. J Psychosom Res.

[8] Toner BB, Stuckless N, Ali A, Downie F, Emmott S, Akman D (1998). The development of a cognitive scale for functional bowel disorders. Psychosom Med.

[9] Sarter L, Heider J, Kirchner L, Schenkel S, Witthöft M, Rief W (2021). Cognitive and emotional variables predicting treatment outcome of cognitive behavior therapies for patients with medically unexplained symptoms: a meta-analysis. J Psychosom Res.

[10] Lackner JM, Jaccard J, Radziwon CD, Firth RS, Gudleski GD, Hamilton F (2019). Durability and decay of treatment benefit of cognitive behavioral therapy for irritable bowel syndrome: 12-month follow-up. Am J Gastroenterol.

[11] Hesser H, Hedman-Lagerlöf E, Lindfors P, Andersson E, Ljótsson B (2021). Behavioral avoidance moderates the effect of exposure therapy for irritable bowel syndrome: a secondary analysis of results from a randomized component trial. Behav Res Ther.

[12] Billones R, Saligan L (2020). What works in mindfulness interventions for medically unexplained symptoms? A systematic review. Asian Pac Isl Nurs J.

[13] Ljótsson B, Hesser H, Andersson E, Lackner JM, El Alaoui S, Falk L (2014). Provoking symptoms to relieve symptoms: a randomized controlled dismantling study of exposure therapy in irritable bowel syndrome. Behav Res Ther.

[14] Craske MG, Wolitzky-Taylor KB, Labus J, Wu S, Frese M, Mayer EA (2011). A cognitive-behavioral treatment for irritable bowel syndrome using interoceptive exposure to visceral sensations. Behav Res Ther.

[15] Kawanishi H, Sekiguchi A, Funaba M, Fujii Y, Yoshiuchi K, Kikuchi H (2019). Cognitive behavioral therapy with interoceptive exposure and complementary video materials for irritable bowel syndrome (IBS): protocol for a multicenter randomized controlled trial in Japan. Biopsychosoc Med.

[16] Bonnert M, Olén O, Lalouni M, Benninga MA, Bottai M, Engelbrektsson J (2017). Internet-delivered cognitive behavior therapy for adolescents with irritable bowel syndrome: a randomized controlled trial. Am J Gastroenterol.

[17] Hunt M, Miguez S, Dukas B, Onwude O, White S (2021). Efficacy of Zemedy, a mobile digital therapeutic for the self-management of irritable bowel syndrome: crossover randomized controlled trial. JMIR MHealth UHealth.

[18] Lalouni M, Ljótsson B, Bonnert M, Ssegonja R, Benninga M, Bjureberg J (2019). Clinical and cost effectiveness of online cognitive behavioral therapy in children with functional abdominal pain disorders. Clin Gastroenterol Hepatol.

[19] Owusu JT, Sibelli A, Moss-Morris R, van Tilburg MAL, Levy RL, Oser M. A pilot feasibility study of an unguided, internet-delivered cognitive behavioral therapy program for irritable bowel syndrome. Neurogastroenterol Motil. 2021:e14108. 10.1111/nmo.14108 33745228

[20] Hughes S, Sibelli A, Everitt HA, Moss-Morris R, Chalder T, Harvey JM (2020). Patients’ experiences of telephone-based and web-based cognitive behavioral therapy for irritable bowel syndrome: longitudinal qualitative study. J Med Internet Res.

[21] Dunlap LJ, Jaccard J, Lackner JM. Minimal-contact versus standard cognitive behavioral therapy for with irritable bowel syndrome: cost-effectiveness results of a multisite trial. Ann Behav Med. 2021:kaaa119. 10.1093/abm/kaaa119 PMC8489303 33821928

[22] Labus JS, Mayer EA, Chang L, Bolus R, Naliboff BD (2007). The central role of gastrointestinal-specific anxiety in irritable bowel syndrome: further validation of the visceral sensitivity index. Psychosom Med.

[23] Labus JS, Bolus R, Chang L, Wiklund I, Naesdal J, Mayer EA (2004). The visceral sensitivity index: development and validation of a gastrointestinal symptom-specific anxiety scale. Aliment Pharmacol Ther.

[24] Hunt MG, Ertel E, Coello JA, Rodriguez L (2014). Development and validation of the GI-cognitions questionnaire. Cognit Ther Res.

[25] Reme SE, Darnley S, Kennedy T, Chalder T (2010). The development of the irritable bowel syndrome-behavioral responses questionnaire. J Psychosom Res.

[26] Drossman DA, Patrick DL, Whitehead WE, Toner BB, Diamant NE, Hu Y (2000). Further validation of the IBS-QOL: a disease-specific quality-of-life questionnaire. Am J Gastroenterol.

[27] Patrick DL, Drossman DA, Frederick IO, DiCesare J, Puder KL (1998). Quality of life in persons with irritable bowel syndrome: development and validation of a new measure. Dig Dis Sci.

[28] Lackner JM, Jaccard J, Krasner SS, Katz LA, Gudleski GD, Holroyd K (2008). Self-administered cognitive behavior therapy for moderate to severe irritable bowel syndrome: clinical efficacy, tolerability, feasibility. Clin Gastroenterol Hepatol.

[29] Leibbrand R, Hiller W (2003). Cognitive behavior therapy for functional gastrointestinal disorders: is group treatment effective?. Acta Neuropsychiatr.

[30] Radu M, Moldovan R, Pintea S, Băban A, Dumitrascu D (2018). Predictors of outcome in cognitive and behavioural interventions for irritable bowel syndrome. A meta-analysis. J Gastrointestin Liver Dis.

[31] Lackner JM, Lou Coad M, Mertz HR, Wack DS, Katz LA, Krasner SS (2006). Cognitive therapy for irritable bowel syndrome is associated with reduced limbic activity, GI symptoms, and anxiety. Behav Res Ther.

[32] Kennedy PJ, Clarke G, Quigley EM, Groeger JA, Dinan TG, Cryan JF (2012). Gut memories: towards a cognitive neurobiology of irritable bowel syndrome. Neurosci Biobehav Rev.

[33] Lackner JM, Jaccard J, Krasner SS, Katz LA, Gudleski GD, Blanchard EB (2007). How does cognitive behavior therapy for irritable bowel syndrome work? A mediational analysis of a randomized clinical trial. Gastroenterology..

